# P-696. A New Approach to Respiratory Infection Surveillance: Weekly Real-Time Analytics of Viral Trends & Healthcare Administration, as a Prelude to VIRAL Network LATAM

**DOI:** 10.1093/ofid/ofae631.892

**Published:** 2025-01-29

**Authors:** Ivan Felipe Gutiérrez Tobar, Carlos Alvarez, Juan Pablo Londono-Ruiz, Adriana Cuenca, Guillermo Eljadue, Alexandra Jimenez, Martha Hernandez, Juan Bravo, Sayonara Florez, Mauricio Pinto, Carlos Arteta, Olga Baquero

**Affiliations:** Clinica Infantil Colsubsidio, Clínica Infantil Santa María del Lago, Bogotá, Distrito Capital de Bogota, Colombia; Departamento Enfermedades Infecciosas, Clínica Colsanitas, Universidad Nacional de Colombia, BOGOTA, Distrito Capital de Bogota, Colombia; Hospital universitario Mayor Mederi, Bogota, Distrito Capital de Bogota, Colombia; Clinica Infantil Colsubsidio, Bogota, Distrito Capital de Bogota, Colombia; Clinica Infantil Colsubsidio, Bogota, Distrito Capital de Bogota, Colombia; Clinínica Infantil Colsubsidio, Bogotá, Distrito Capital de Bogota, Colombia; CLINICA INFANTIL SANTA MARIA DEL LAGO, BOGOTA, Distrito Capital de Bogota, Colombia; CLINICA INFANTIL SANTA MARIA DEL LAGO, BOGOTA, Distrito Capital de Bogota, Colombia; Clínica Infantil Santa María del Lago, Bogota, Distrito Capital de Bogota, Colombia; Clínica Infantil Santa María del Lago, Bogota, Distrito Capital de Bogota, Colombia; Clínica Infantil Colsubsidio, Bogota, Distrito Capital de Bogota, Colombia; Clínica Infantil Colsubsidio, Bogota, Distrito Capital de Bogota, Colombia

## Abstract

**Background:**

The circulation of respiratory viruses varies depending on the region, impacting local and regional hospitals as well as public health. This article describes the experience of implementing a system to monitor weekly how changes in viral isolations affect hospital management at a pediatric reference center.

A. Variation of the 5 most common pathogens in the first 18 weeks of 2024. and B. Correlation between the number of tests conducted and the frequency of viral isolates.

**Figure:**
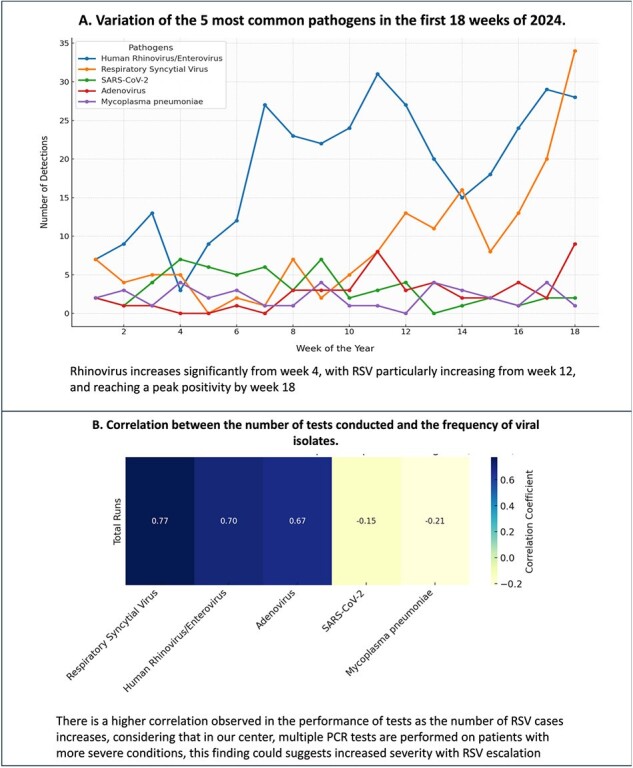

A. Rhinovirus increases significantly from week 4, with RSV particularly increasing from week 12, and reaching a peak positivity by week 18

B. There is a higher correlation observed in the performance of tests as the number of RSV cases increases, considering that in our center, multiple PCR tests are performed on patients with more severe conditions, this finding could suggests increased severity with RSV escalation

**Methods:**

An ecological study, utilized molecularly identified viral isolations and ICD-10 coded Hospital Admissions Database entries, integrated with weekly consolidated data on antimicrobial stewardship, respiratory therapy, and hospital indicators. Using Python and R, we developed matrices to trace viral variation patterns and their impact on hospital management. Descriptive and correlation analyses explored potential links between viral frequency and hospital metrics in pediatric emergency and hospitalization settings, shedding light on the nuanced dynamics of hospital responses.

A. Frequency of the 5 most common pathogens and their relationship with the number of cases diagnosed with asthma in pediatric emergency room and B. Correlation between the number of asthma cases in pediatric emergencies and the number of viral pathogen isolations.

**Figure:**
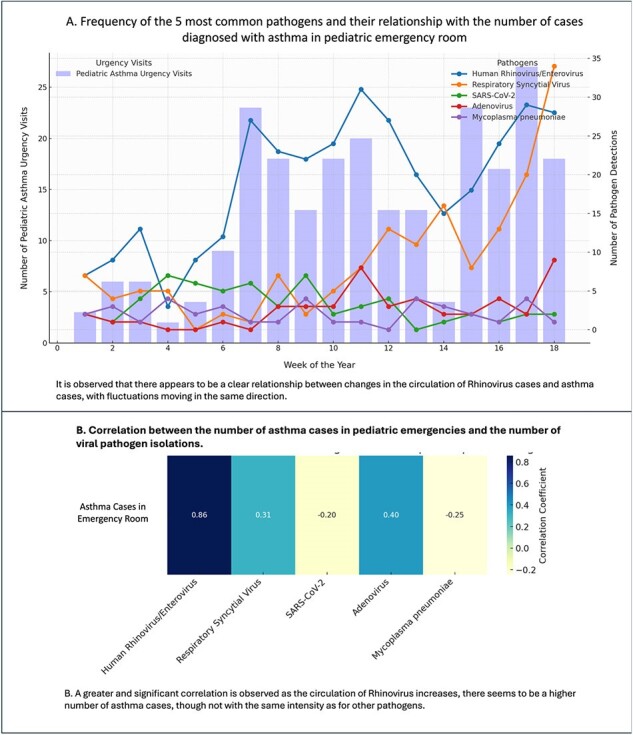

It is observed that there appears to be a clear relationship between changes in the circulation of Rhinovirus cases and asthma cases, with fluctuations moving in the same direction.

B. A greater and significant correlation is observed as the circulation of Rhinovirus increases, there seems to be a higher number of asthma cases, though not with the same intensity as for other pathogens.

**Results:**

By week 18 of 2024, we conducted 675 diagnostic tests, with 85.63% positivity rate. Additionally, we analyzed 583 pediatric hospitalizations and 1229 ER visits for ARI. Notably, Rhinovirus cases showed a significant increase from week 6, surpassing other common pathogens, reflecting a similar trend seen in asthma cases. Moreover, from week 12, there was a rise in RSV positivity, peaking in week 18. With the heightened circulation of Rhinovirus and RSV in the pediatric clinic, there was an observable shift in the frequency of antibiotic prescriptions for ARI compared to other infections. The surge in RSV cases appears to correlate with severity, as indicated by the increased number of tests conducted on patients with significant respiratory compromise in our center.

Percentage of Antibiotics Used Through Week 17 at a Reference Pediatric Center (Acute Respiratory infection vs Non-ARI) and Variation in Most Frequent Respiratory Pathogens

**Figure:**
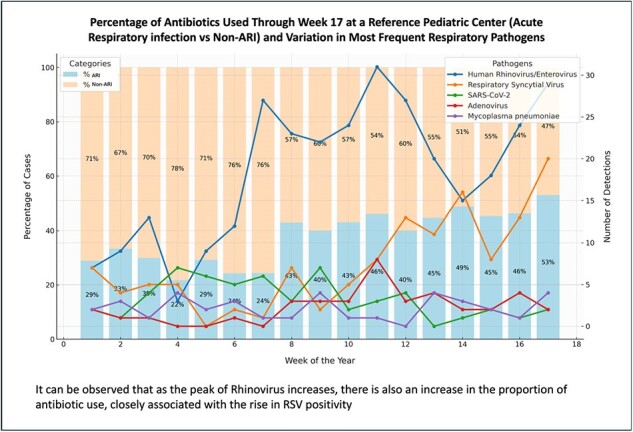

It can be observed that as the peak of Rhinovirus increases, there is also an increase in the proportion of antibiotic use, closely associated with the rise in RSV positivity

**Conclusion:**

The viral circulation variation alters hospital management behavior. With increased Rhinovirus cases, there appears to be a heightened association with asthma, and as RSV cases rise, there's an apparent increase in severity and the number of related tests. Antibiotic prescription percentages in the clinic are influenced by viral circulation fluctuations. This surveillance model provides insights useful for identifying unusual behaviors, improving respiratory peak anticipation and management, and quantifying the true burden of viral circulation and potential prevention strategy impacts.

**Disclosures:**

**All Authors**: No reported disclosures

